# Effects of Connectivity and Recurrent Local Disturbances on Community Structure and Population Density in Experimental Metacommunities

**DOI:** 10.1371/journal.pone.0019525

**Published:** 2011-04-29

**Authors:** Florian Altermatt, Annette Bieger, Francesco Carrara, Andrea Rinaldo, Marcel Holyoak

**Affiliations:** 1 Department of Aquatic Ecology, Eawag: Swiss Federal Institute of Aquatic Science and Technology, Dübendorf, Switzerland; 2 Department of Environmental Science and Policy, University of California Davis, Davis, California, United States of America; 3 Laboratory of Ecohydrology, Ecole Polytechnique Fédérale de Lausanne, ENAC School of Architecture, Civil and Environmental Engineering, Bâtiment GR, Lausanne, Switzerland; University of Montpellier II, France

## Abstract

Metacommunity theory poses that the occurrence and abundance of species is a product of local factors, including disturbance, and regional factors, like dispersal among patches. While metacommunity ideas have been broadly tested there is relatively little work on metacommunities subject to disturbance. We focused on how localized disturbance and dispersal interact to determine species composition in metacommunities. Experiments conducted in simple two-patch habitats containing eight protozoa and rotifer species tested how dispersal altered community composition in both communities that were disturbed and communities that connected to refuge communities not subject to disturbance. While disturbance lowered population densities, in disturbed patches connected to undisturbed patches this was ameliorated by immigration. Furthermore, species with high dispersal abilities or growth rates showed the fastest post-disturbance recovery in presence of immigration. Connectivity helped to counteract the negative effect of disturbances on local populations, allowing mass-effect-driven dispersal of individuals from undisturbed to disturbed patches. In undisturbed patches, however, local population sizes were not significantly reduced by emigration. The absence of a cost of dispersal for undisturbed source populations is consistent with a lack of complex demography in our system, such as age- or sex-specific emigration. Our approach provides an improved way to separate components of population growth from organisms' movement in post-disturbance recovery of (meta)communities. Further studies are required in a variety of ecosystems to investigate the transient dynamics resulting from disturbance and dispersal.

## Introduction

Metacommunities are defined by dispersal connecting local communities of potentially interacting species [1], [2]. Consequently, diversity and abundance of species in a spatially-structured region is determined by both local and regional processes. Dispersal into a patch may increase local population sizes by mass or rescue effects, or augment diversity through immigration of new species [1]. From the perspective of the community of origin, however, dispersal also causes a loss of individuals [3], [4], [5]. There are few empirical studies of the effect of such loss on local populations and communities because of the difficulties in manipulating dispersal and observing whole communities.

In many natural systems, the onset of emigration from a patch is triggered by characteristics of the donor population as well as by local environmental conditions in that patch [6], [7], [8], [9]. Among environmental factors, disturbance is thought to strongly affect dispersal [10], [11], [12], [13], [14] and metacommunity theory offers an ideal concept to study disturbances in a spatial context [1], [15]. Many disturbances are highly stochastic, and create both temporal and spatial variability in usable habitat patches [16], and may even increase fragmentation and reduce connectivity [17]. Disturbances such as floods or fires have, by definition, an initial negative effect on existing local communities [16]. Disturbances can also be a regional structuring force, for example when landslides in the headwater of a stream affect long stretches of fluvial habitats [15]. Localized disturbances, which are the focus of this study, may initiate the dispersal of individuals away from disturbed patches [10], [13]. However, disturbances also free resources and open niche space on a local scale [16]. After a disturbance, resources in a local patch can thus be exploited by locally surviving individuals, but also by individuals arriving from other patches. Thereby, the effects of a local disturbance might go far beyond the local patch [4]: either by triggering increased dispersal from disturbed into undisturbed patches, or conversely by triggering dispersal from undisturbed to disturbed patches. In both cases, the immigrating individuals interact with local resident individuals of a variety of species, and may change local community composition. Immigration may also enrich local communities through introduction of resources, adding new individuals that can then exponentially multiply in times of abundant resources, or through introducing genetic diversity [7].

A recent theoretical study showed that asymmetric dispersal in a two-patch metacommunity affected community composition by promoting coexistence of competing species [18]. In heterogeneous metacommunities without asymmetric dispersal, however, dispersal may decrease diversity by enhancing regional competition [19], [20], [21]. Furthermore, the indirect effects of disturbances and subsequent emigration or immigration on community composition in metacommunities may lead to non-linear dynamics, for example when populations recover from disturbances by a combination of reproduction by local survivors and immigrants or by altering among-species interactions [22]. Altered species interactions may be especially relevant in models of competitive metacommunities [22], [23], in which a competition-colonization trade-off can explain species coexistence [24], [25]. Nonlinearities resulting from such species interactions might make recovery from disturbances in metacommunities give some unexpected effects. A variety of empirical studies have investigated source-sink dynamics and how they affect competitive outcomes or predator-prey dynamics [26], [27]. Empirical studies also have shown that species diversity and diversification may not only be influenced by the spatial direction of dispersal, but also by the temporal sequence of immigration which can cause priority effects [28], [29], [30]. This may be especially relevant following recurrent disturbance. Additionally, recent empirical studies have started to consider the effect of post-disturbance recovery on the surrounding habitat matrix. For example, Brudvig et al. [31] found that habitat corridors connecting patches of forest clearings not only facilitate movement of organisms between patches, but additionally benefit plant-diversity in surrounding non-target habitats in a so-called biodiversity spillover effect [32], [33]. There is, however, no general conclusion about how disturbance-induced changes in population or community composition affect adjacent communities.

We studied how recurrent, local disturbances and connectivity of patches in metacommunities affect species richness and abundance. We addressed three specific questions: (1) Does connectivity between disturbed and undisturbed patches lead to a net immigration to disturbed patches, and if so, does this increase the rate of post-disturbance recovery? (2) Does disturbance in adjacent patches reduce diversity or abundance in undisturbed patches? (3) Which (if any) species traits explain post-disturbance recovery of populations?

We addressed these questions using microcosm experiments with protists and rotifers. This system has been used in other empirical studies, which have focussed on localized disturbances [34], [35], [36]. In these studies, disturbance of patches occurred randomly, such that each patch within a metacommunity had the same likelihood of being disturbed, and dispersal was manipulated by manually moving individuals among patches. In nature, however, disturbances are often temporally or spatially aggregated [16], [37], [38], and dispersal occurs naturally and post-disturbance recovery of local communities involves a combination of population growth and movement. Here, we used simple metacommunities consisting of just two patches, one of which was subjected to recurrent local disturbance. Dispersal between the two patches occurred naturally by movement of individuals through a corridor. Such two-patch systems facilitate the study of mechanisms because they excluded complex interactions such as distance-dependent dispersal or effects from the spatial arrangement of more than two patches. Consequently, they have been widely used in theoretical models to study dynamical mechanisms of species coexistence and diversity in patchy landscapes [18], [39], [40].

We expected that species with a high dispersal ability may rapidly recolonize disturbed patches from undisturbed ones, and they therefore should recover from disturbances more quickly than species with low dispersal rates. Mass-effects of species with large population sizes may cause similar patterns to those expected from high per capita dispersal abilities. However, population recovery from individuals surviving disturbance could also occur within patches, and might be important for species with a high growth rate. Furthermore, we expect that by separating traits that directly relate to organismal movement from those quantifying population growth we can improve identification of the role of species traits in (meta)community dynamics, especially post-disturbance recovery.

## Materials and Methods

We conducted our experiment in aquatic microcosms containing seven protozoan species, one rotifer species and a set of common freshwater bacteria as a food resource. Bacteria, in turn were supported on a plant-based nutrient medium and decomposing wheat seeds. The seven protozoan species were *Chilomonas* sp., *Colpidium* sp., *Euglena gracilis*, *Euplotes aediculatus*, *Paramecium aurelia*, *P. bursaria* and *Spirostomum* sp., while the rotifer remained unidentified (cf. *Rotifera* sp.). Five of the protozoan and the rotifer species we studied were originally collected from a single pond [41], while *Chilomonas* sp. and *Spirostomum* sp. came from Carolina Biological Supply Company, Burlington, NC, USA. All species are predominantly bacterivores, although some may also consume on other (smaller) protozoans and *Eug. gracilis*, *Eup. aediculatus* and *P. bursaria* can also photosynthesize. The herein used microcosms are a simple biological system and offer a useful bridge between theory and empirical tests in nature. They have been used by our own and other labs to experimentally address questions in community and metacommunity biology, with some focus on dispersal and disturbance [24], [26], [35], [42], [43], [44], [45], [46], [47].

### Experimental set-up

**Figure 1:**
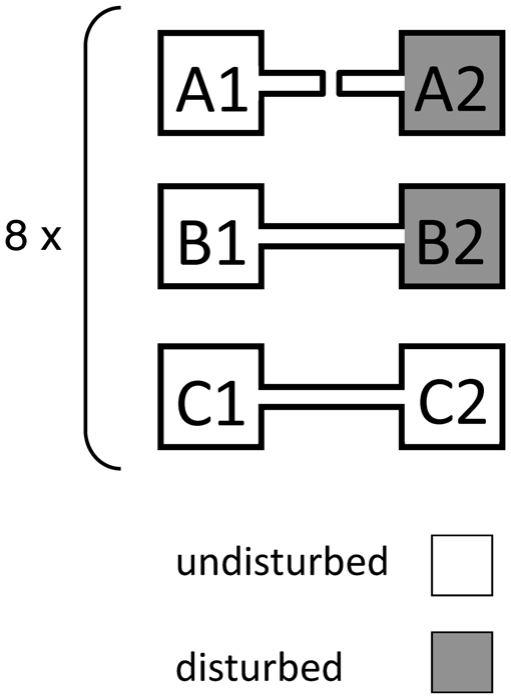
Set-up of the experimental microcosms. We had three different types of metacommunities (A, B and C), each consisting of two patches and all eightfold replicated. Metacommunities of type A consisted of two isolated patches, one of which (A2) was regularly disturbed, while the other (A1) was undisturbed. Metacommunities of type B consisted of two connected patches, one of which (B2) was regularly disturbed, while the other (B1) was undisturbed. Metacommunities of type C consisted of two connected patches (C1 and C2), which were both undisturbed.

We used three different types of metacommunities (A, B, and C; Fig. 1): In metacommunities of type A, the two patches were unconnected and no dispersal occurred. One randomly-chosen patch experienced recurrent disturbances (A2) while the other patch did not (A1). In metacommunities of types B and C, the two patches were connected and individuals could disperse naturally in both directions between the two patches (between B1 and B2 or C1 and C2). In metacommunities of type B, one randomly-chosen patch experienced recurrent disturbance (B2) while the other patch did not (B1), and the occurrence of disturbance was the same as metacommunities of type A. In metacommunities of type C, both patches remained undisturbed. Each treatment was replicated 8 times, resulting in a total of 24 metacommunities. We know from previous work that the species were able to coexist in undisturbed communities [42], [43], and that dispersal occurs in such a setup [24], [42], [43].

Like in Davies et al. [42], each of the individual patches in a microcosm consisted of a 125-ml Nalgene square Polycarbonate wide-mouth bottle. The two bottles of a metacommunity were connected with 12.7 cm of silicon tubing (inner diameter 6.4 mm). To control for effects of tubing such as spatial refuge or spatial heterogeneity, we furnished the isolated controls with equal length of tubing but clamped off the centre of the tubing. Each bottle was filled with 100 ml of nutrient medium and two autoclaved wheat seeds as an additional carbon source for the bacteria. The medium was a standard soil-water solution, prepared by mixing 2.4 g of sterilized soil, 0.6875 g of Protozoan Pellet (Carolina Biological Supply Co.), and 0.1 g of Herptivite multivitamin mixture (Rep-Cal Research Labs Company, CA, USA) in 1.5 l of spring water and then sterilized by steam autoclaving. A day before adding the protozoa and rotifer species, this solution was inoculated with 1 ml of a mixed bacterial culture to provide resources for protozoans. The culture consisted of *Bacillus cereus*, *B. subtilis*, and *Serratia marcescens* obtained from Carolina Biological Supply Company. All bottles were loosely capped during the whole experiment to minimize evaporation, and sterile technique was used throughout the experiment. Each bottle was initiated with a community of all eight protozoa or rotifer species. The inoculum containing the species was added by volume (after removing an equivalent volume of nutrient medium). Initial population numbers per bottle were set to about 100 individuals for all species but *Spirostomum* sp., which naturally occurs at lower densities than the other species and was initiated with a population of about 30 individuals per bottle. Initial population numbers were set to avoid extinction caused by demographic stochasticity before application of the first disturbance treatment. All species were capable of persisting at these starting densities, as demonstrated by their persistence in the undisturbed and isolated controls. All communities were allowed to grow for one week before disturbance treatments were applied.

Disturbance consisted of replacement of 99% of the bottle contents with sterilized media. Similar disturbance treatments have been extensively explored in previous studies [36], [43], [45], but (with the exception of [45]) were done in a non-spatial context. Disturbances did not affect the c. 4 ml of medium in the tubing of the dispersal-corridor, in which protozoa could survive, just as dispersing individuals in a habitat matrix would not experience patch-specific disturbance in nature. We always disturbed the same microcosm bottle (patch 2) within a metacommunity, and disturbances occurred every 3–4 days (every Monday and Thursday). In total we had 11 disturbance events over the whole experimental period of 43 days. We clamped the tubing between two bottles prior to the disturbance treatment to minimize interpatch movement of organisms caused by handling. To prevent population collapse due to nutrient depletion, we replaced 10 ml of microcosm contents with sterile medium in each undisturbed community after 3 weeks [43]; we did not conduct this procedure in disturbed patches because nutrient medium was already being replaced through the disturbance process.

We estimated the density (and presence/absence) of the protist and rotifer study species in each replicate with a stereo-microscope (20–40× magnification) after 32 days and after 43 days ( = first and second sampling, respectively). Samples were taken two days after disturbance events. We thoroughly mixed the contents of each bottle prior to the sampling, and took a sub-sample of 10 ml. After the first sampling, the bottles were refilled with an equal amount of sterile medium. Because of the different sizes and densities of species, volumes censused (from the 10 ml sample) were species-specifically adjusted to obtain an adequate density estimate [42], [43].

We compared species-specific differences in densities between the different treatments with three species-specific traits, and a fourth parameter, which was an integrated measurement of the predicted rate of spread. The three species-specific traits were intrinsic growth rate (*r*), carrying capacity (*K*), and velocity (*v*). These independent measures provided a method for separating the role of between-patch movement from subsequent population growth. For most species, estimates of growth rate and carrying capacity were available from published work [43], [48]. For *Chilomonas* sp. and the rotifer species, however, appropriate published estimates of *r* and *K* were not available. We thus determined *r* and *K* for these two species using the methods of Haddad et al. [43]. In our experiment, we had only one nutrient level, which was equivalent to the high-nutrient level in Haddad et al. [43] and which was identical to the medium used in all other experiments of our study. We separately conducted single species growth experiments, and cultures were prepared as in the main experiment. For each of these two species, we prepared 5 bottles, filled with 100 ml of the soil–water solution. Treatments were started with c. 10 individuals per ml. We measured densities in cultures of ages 0.5, 1, 2, 3, 4, 7, and 14 days, by which time each species had reached or surpassed its carrying capacity, estimated as the long-term equilibrium density. We counted densities as in the main experiment. Estimates were generated for each of 5 replicate nutrient treatments. In each case, logistic growth was supported over exponential or theta-logistic growth. The final estimates of *r* and *K* were the average of the values generated from the 5 replicates; for details of the method see [43]. To obtain an estimate of movement we measured the swimming speed (velocity) of each of the eight species. We separately placed 4.75 ml medium from cultures at carrying capacity in a glass-petri dish of 11 cm diameter. We then measured the maximum speed of 40–50 randomly chosen individuals per species. Measurements were taken with an Olympus SZX16 microscope and a digital Olympus DP72 camera. We tracked straight-swimming individuals at maximum swimming-speed with the image analysis software cell∧D (version 3.2). Magnification used and time-intervals measured depended on the species' behaviour (between 0.25 to 1.5 s was chosen to obtain the maximum length of straight swimming of each species). Moreover, we did not measure velocity of individuals while they were feeding or changing direction. We measured swimming distances in µm and calculated mean velocity (swimming speed) for each species in µm s^−1^. To study post-disturbance recolonization of disturbed habitat patches it is more relevant to consider the spatial spread of an organism rather than just its ability to move. From a theoretical point of view, the integrated spread of organisms can often be described with travelling waves, using reaction-diffusion transport-models [49], [50]. If the spread undergoes a diffusion process, the minimum speed of the travelling wave is then determined by the organisms' intrinsic growth rate *r* and a species-specific diffusion coefficient *D* [51]. Consequently, the speed of the wavefront (analogous to the front of dispersal) can be calculated from population parameters, using *r* and *v* to get an indirect measurement for *D*. *D* is proportional to 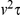, 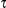 being the mean time in between two changes of direction of a moving individual and assumed, in a first approximation, as a constant for the species. Thus, the predicted rate of spread is then proportional to 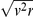 [52].

### Analyses

We conducted a series of planned contrasts for local species richness. We compared local species richness in isolated, disturbed communities (A2; Fig. 1) with local species richness of disturbed patches (B2), which were connected to undisturbed patches, using a repeated-measures ANOVA (question 1). We used treatment as a fixed effect and introduced an error term for the two sequential time-points when each population was sampled. We then compared local species richness of undisturbed, isolated patches with undisturbed patches that where either connected to disturbed or undisturbed patches (comparing A1, B1 and C1) using the same repeated measures ANOVA model as described above (question 2).

We correlated the local population density in each patch and for each species at the first and the second sampling. Furthermore, we compared the local densities of all species in isolated disturbed patches (A2; Fig. 1) with local densities in disturbed patches (B2), which were connected to undisturbed patches (using a MANOVA). Repeated measures analyses were not possible with MANOVA's. We thus analysed the first and second sampling separately and repeated the analysis on time-averaged densities (question 1). We also compared densities of all species in undisturbed, isolated patches with undisturbed patches that were either connected to disturbed or undisturbed patches (comparing A1, B1 and C1, again using a MANOVA for the first sampling, the second sampling and time-averaged densities; as in question 2). To fulfil normality assumptions, we log_10_-transformed the density data. We excluded *Chilomonas* sp. from the comparison of undisturbed communities and *Spirostomum* sp. from the comparison of disturbed communities, because these species went extinct under the respective treatment regimes, and hence no density estimates were available.

Finally, we compared species-specific differences in mean densities at the second sampling between different treatments and the species' traits with a linear correlation (Pearson's coefficient; question 3). This tested whether species-specific traits could explain density differences between isolated, undisturbed communities and isolated disturbed communities (A1–A2), or between connected, undisturbed communities and connected, disturbed communities (B1–B2), between isolated, disturbed communities and connected disturbed communities (A2–B2). All analyses were conducted using *R* [53].

## Results

**Figure 2:**
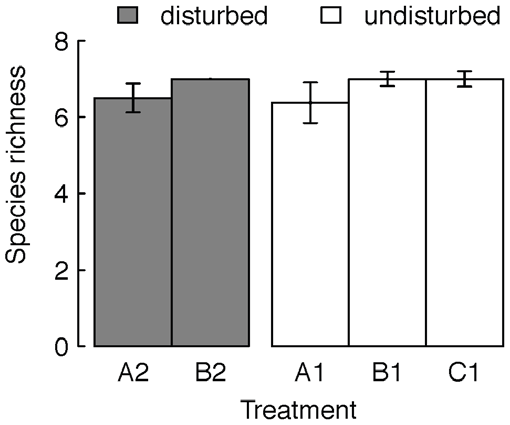
Mean species richness at the first sampling (32 days). Mean species richness (±se) within single communities in undisturbed and disturbed patches (white and grey bars respectively). A1 and A2 were isolated patches, while B1, B2 and C1 were connected patches (see also Fig. 1 and 2).

**Figure 3:**
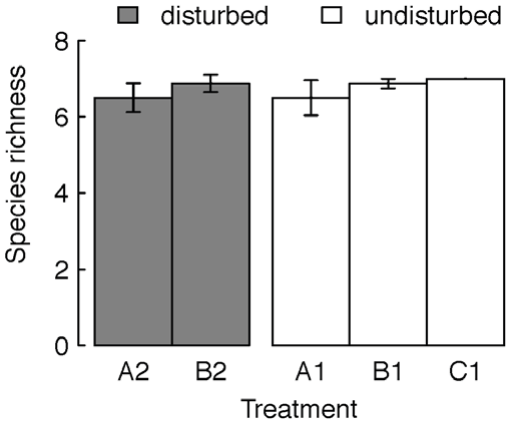
Mean species richness at the end of the experiment. Mean species richness (±se) within single communities in undisturbed and disturbed patches (white and grey bars respectively). A1 and A2 were isolated patches, while B1, B2 and C1 were connected patches (see also Fig. 1).

Mean local species richness remained consistently high in all patches (Fig. 2,3), with on average 81–88% of the initial species being locally present at the end of the experiment (Fig. 3). Local species richness of isolated, disturbed communities was not significantly different from local species richness of disturbed patches, which were connected to undisturbed patches and there was no significant effect of the time of sampling on species richness (local species richness of A2 vs. B2; repeated measures ANOVA: treatment, F_1,14_ = 1.23, p = 0.29; sampling time, F_1,15_ = 0.32, p = 0.58; Fig. 3). Also, local species richness of undisturbed patches was not significantly affected by being connected to other patches or by the occurrence of disturbances in the patch they were connected with (comparing local species richness of A1, B1 and C1; repeated measures ANOVA: treatment, F_2,20_ = 0.99, p = 0.39; sampling time, F_2,22_<0.001, p = 1; Fig. 3). Local species richness was slightly lower at the second time of sampling (Fig. 3) compared to the first sampling (Fig. 2), but not significantly different.

**Figure 4:**
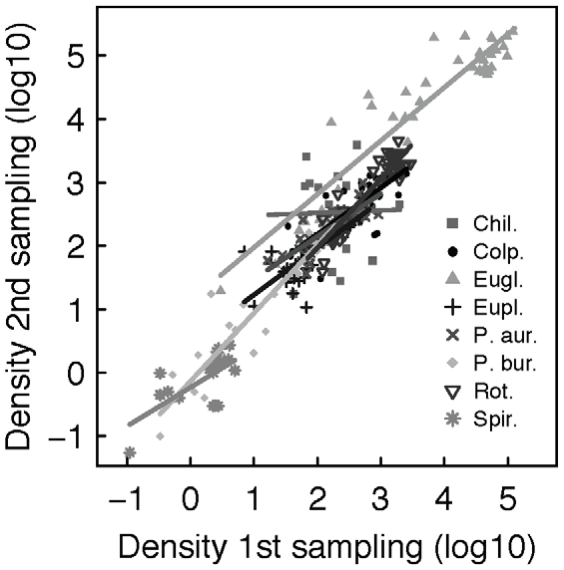
Population densities after 32 and 43 days. Species-specific linear correlations between the density in the first and second sampling, after 32 and 43 days respectively (density data log_10_-transformed; for statistics see also table 1). In all but one species (Chi), density at the second sampling was strongly correlated with density at the first sampling. Abbreviations of the species: Chil.  =  *Chilomonas* sp., Colp. *Colpidium* sp., Eupl.  =  *Euplotes aediculatus*, P. aur.  =  *Paramecium aurelia*, P. bur.  =  *Paramecium bursaria*, Rot.  =  rotifer, and Spir.  =  *Spirostomum* sp.

**Figure 5:**
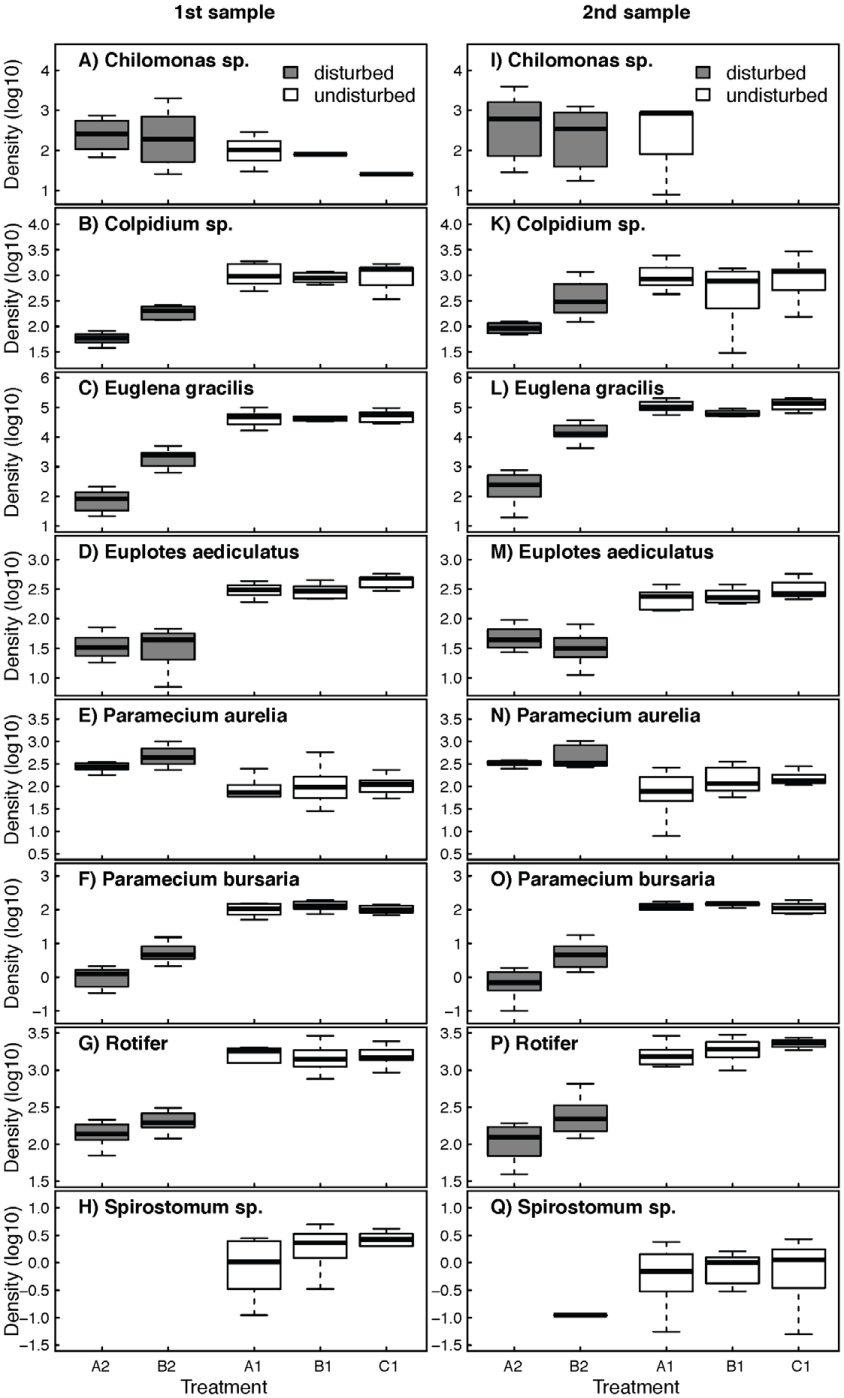
Population densities of all protozoa and rotifer species. Density (log_10_) of all eight species within single communities in undisturbed and disturbed patches (white and grey boxplots respectively) after 32 days (first sampling; A–H) and at the end of the experiment after 43 days (I–Q). A1 and A2 were isolated patches, while B1, B2 and C1 were connected patches (see also Fig. 1). Boxplots give median (bold line), first and third quartile (box). Whiskers give either the range of the data or 1.5 times the interquartile range, whichever is smaller.

Local densities at the second sampling were for all but one species (*Chilomonas* sp.) highly significantly correlated with local densities at the first sampling (Fig. 4 and Table 1). Contrary to local species richness, we found a significant effect of connectivity and disturbance on local species density. At the first sampling after 32 days, local density of species in isolated, disturbed communities was significantly lower than that in disturbed patches connected to undisturbed patches (species-specific local densities in A2 vs. B2; MANOVA, Pillai = 0.96, F_6,7_ = 22.3, p<0.001; Fig. 5). This effect was also found at the end of the experiment after 43 days. Again, local density in isolated, disturbed communities was significantly lower compared to local density in disturbed patches, which were connected to undisturbed patches (MANOVA, Pillai = 0.95, F_7,4_ = 11.6, p = 0.016; Fig. 5). Consistently, the effect was also found for time-averaged density-estimates (MANOVA, Pillai = 0.99, F_7,2_ = 85.8, p = 0.012). The density of four species was significantly higher in the disturbed, connected patch compared the disturbed, isolated patch: in *P. bursaria* the density difference was 5-fold (first sampling) to 7-fold (second sampling) higher in the connected patch, in *Colpidium* sp. and the rotifer species 1.5 to 2.5-fold and in *Eug. gracilis* 25-fold and to 13-fold respectively. *Chilomonas* sp., *P. aurelia* and *Eup. aediculatus* were not significantly affected by connectivity and disturbance. No comparison of densities could be made for *Spirostomum* sp., because it went extinct under one treatment regime (A2), and hence no density estimates were available.

**Table 1:** Species-specific linear correlations between the density of each species in the first and second sampling after 32 and 43 days respectively (density data log_10_-transformed; see also Fig. 5).

Species	Estimated intercept	Estimated slope	Adj. r^2^	F-value	P-value
*Chilomonas sp.*	2.4423	0.0396	−0.0708	0.009	0.928
*Colpidium sp.*	0.7137	0.7349	0.6633	75.85	1.72e–10
*Euglena gracilis*	1.1349	0.8400	0.8759	269.1	2e–16
*Euplotes aediculatus*	0.4868	0.7415	0.6903	79.02	2.17e–10
*Paramecium aurelia*	0.8009	0.6751	0.6715	72.54	6.00e–10
*Paramecium bursaria*	−0.1364	1.0776	0.9059	328.5	<2e–16
*Rotifer*	−0.2098	1.0903	0.8602	234.9	<2e–16
*Spirostomum sp.*	−0.2328	0.6121	0.4216	14.12	0.0016

Species-specific density in undisturbed patches, on contrary, was not significantly affected by the connectivity of the undisturbed patch or by the occurrence of disturbances in the neighbouring patch (comparing local species-specific density of patches A1, B1 and C1) neither at the first (MANOVA, Pillai = 0.87, F_14,18_ = 0.99, p = 0.50) nor the second sampling (MANOVA, Pillai = 0.79, F_14,20_ = 0.93, p = 0.55; Fig. 5). Consistently, there was no significant effect when using time-averaged density-estimates (MANOVA, Pillai = 0.69, F_14,24_ = 0.91, p = 0.56). *Chilomonas* sp. was not included in that analysis, because it went extinct under two treatment regimes (B1 and C1), and hence no density estimates were available. To ensure that our analysis was powerful enough to detect differences, we repeated the MANOVA, but incrementally reduced the density data in one patch type (B1), while keeping the variance as in the raw data. At a density reduction of 30% or more, the p-value became <0.05 (data not shown), showing that our approach was powerful enough to detect density differences that were much smaller than found in comparison of disturbed, connected and disturbed, isolated communities.

**Figure 6:**
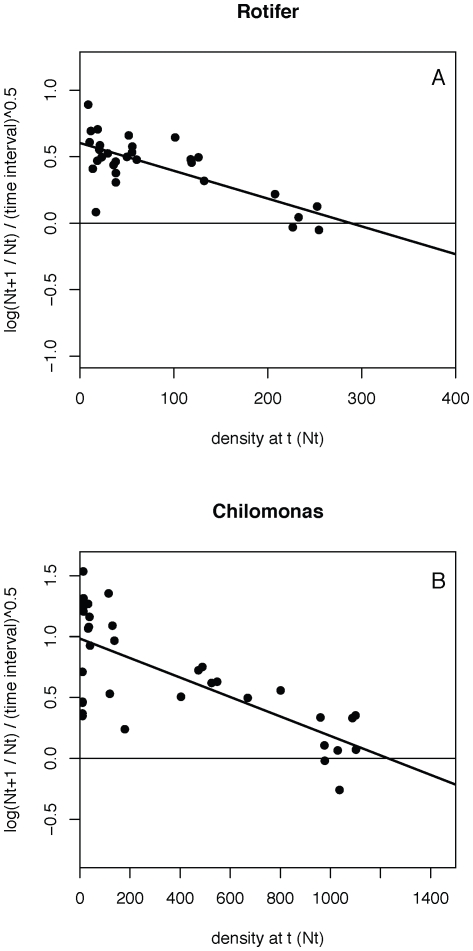
Relationship between population size and growth rate for *Chilomonas* sp. (A) and the rotifer species (B). Black points show growth rates from 5 replicates each at high nutrients microcosms as generally used in our study. Lines show the best fit estimates derived for *r* and *K*. Although we show all points, *r* and *K* were determined separately for each microcosm and then averaged to generate the best fit line.

**Figure 7:**
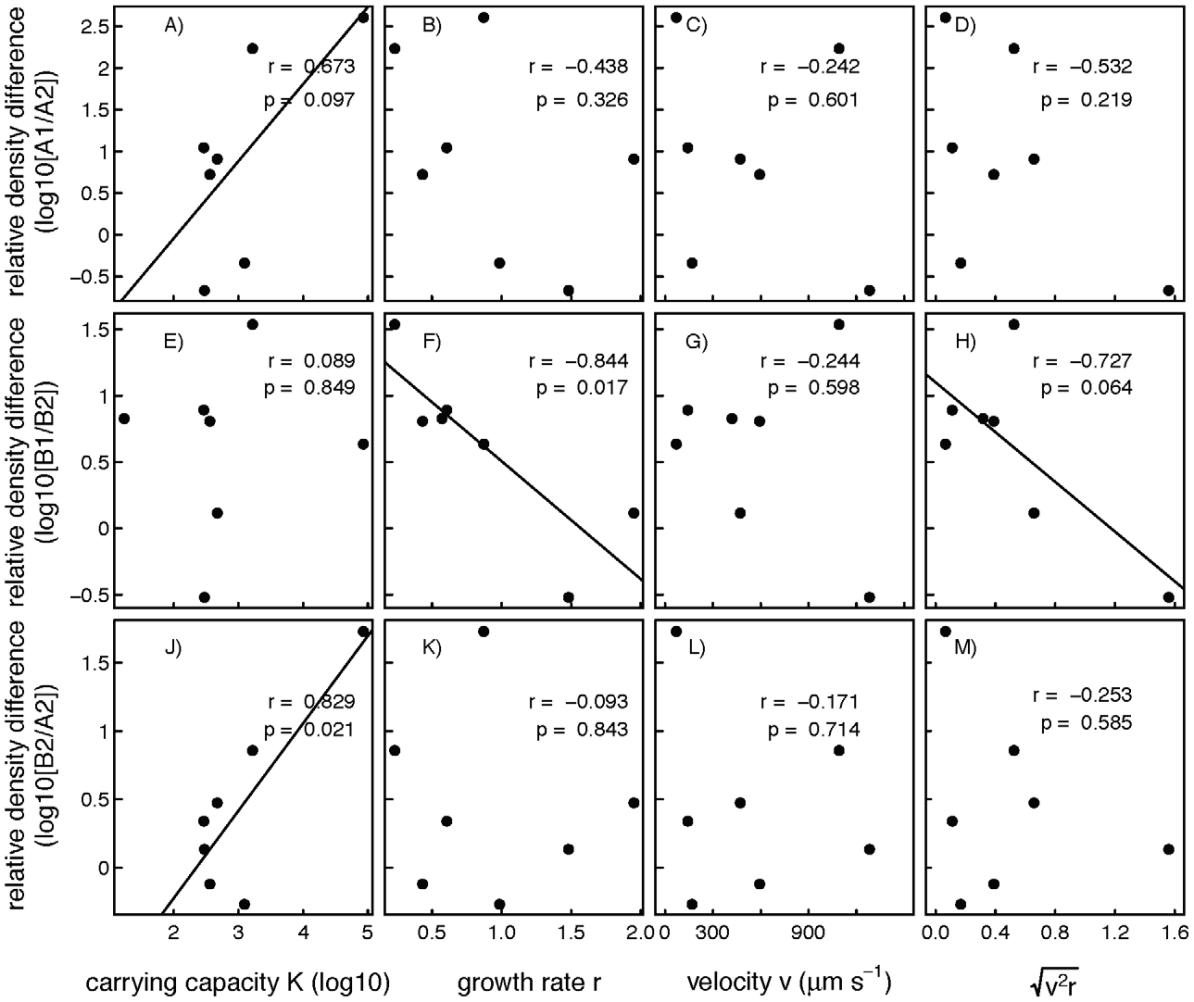
Relationship between species traits and species-specific differences in density between communities experiencing different treatments. Each point stands for a different species. Treatments follow Fig. 1. For species that went extinct in one treatment, values could not be calculated. The predicted rate of spread (D, H, J) is calculated from data on growth rate and velocity. For relationships with p<0.1, the least-square line is given.

The five-replicate averages of intrinsic growth rates and carrying capacities of *Chilomonas* sp. and rotifer sp. were as follows (all measured at the same nutrient levels as in the main experiment): *Chilomonas* sp. *r* = 0.984, *K* = 1232 Ind. ml^−1^; rotifer sp. *r* = 0.604, *K* = 289 Ind. ml^−1^ (Fig. 6). Observed mean (±SE) velocity of each species was 168±5 µm s^−1^ for *Chilomonas* sp., 470±12 µm s^−1^ for *Colpidium* sp., 69±2 µm s^−1^ for *Eug. gracilis*, 592±20 µm s^−1^ for *Eup. aediculatus*, 1281±39 µm s^−1^ for *P. aurelia*, 1090±29 µm s^−1^ for *P. bursaria*, 418±18 µm s^−1^ for *Spirostomum* sp. and 141±4 µm s^−1^ for the rotifer. We tested the ability to predict species-specific differences in local density between different treatments (A1–A2; B1–B2; B2–A2) of our three measured species traits (carrying capacity *K*, growth rate *r*, velocity *v*) and a compound parameter that measured the predicted rate of spatial spread (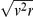; Fig. 7). We found that protozoa and rotifer species with high carrying capacities had marginally significant stronger density reductions in disturbed, isolated patches (A2) compared to undisturbed, isolated patches (A1; r = 0.67, p = 0.097), but the difference in density between patches was not correlated with the species' growth rate, velocity or predicted rate of spread (Fig. 7B to 7D). The species-specific difference in mean density between undisturbed patches (B1) and disturbed but connected patches (B2) was significantly correlated with the species' intrinsic growth rates (r = −0.84, p = 0.017; Fig. 7F) and marginally significantly correlated with the predicted rate of spread (r = −0.73, p = 0.064; Fig. 7F), but not significantly with carrying capacity (Fig. 7E) and species' velocity (Fig. 7G). Finally, carrying capacity was significant in explaining the species-specific difference in density between disturbed, isolated patches (A2), and disturbed, connected patches (B2; r = 0.829, p = 0.021; Fig. 7J); in other words, in disturbed patches populations of species with a high carrying capacity profited most by being connected to undisturbed patches. Again, there was no correlation between growth rate, velocity or predicted rate of spread and the density differences between A2 and B2 (Fig. 7K to 7M).

## Discussion

In experimental metacommunities of protozoa and rotifer species, recurrent disturbances in isolated patches significantly reduced local population densities (Fig. 5) but not species richness (Fig. 2) compared to disturbed patches connected to undisturbed patches. Connectivity between disturbed and undisturbed patches lead to a net immigration to disturbed patches and increased the rate of post-disturbance recovery (question 1). However, disturbance in adjacent patches did not reduce diversity or abundance in undisturbed patches (question 2). Furthermore, we found that even in the absence of local extinctions in the disturbed habitat, dispersal and subsequent rapid intrinsic population growth (but not swimming velocity as an indicator of dispersal ability) hastened population recovery from disturbances when the disturbed patch was connected to an undisturbed patch (question 3).

We found an effect of connectivity and dispersal on density (but not species richness) in disturbed communities connected to undisturbed communities while there was no such effect in disturbed, isolated communities (Fig. 5, treatment A2 vs. B2). Such a rescue or mass effect of connectivity and dispersal on species richness has been demonstrated in other studies [54], [55], [56]. We now demonstrate that it can also occur at the level of population densities. Interestingly, however, the effect was only found in five out of eight species (Fig. 5). In the other three species (*Chilomonas* sp., *Eup. aediculatus* and *Spirostomum* sp.; Fig. 5), the density in disturbed, connected patches was virtually the same as in disturbed, unconnected patches.

Our study provides an important advance over previous studies of the effects of recurrent disturbance [43] through providing a method for separating out the role of between-patch movement from subsequent population growth. Comparison of species traits indicated that post-disturbance recovery in disturbed, connected communities depended mostly on intrinsic growth rate of the species (Fig. 7F), which is expected to be important to recovery from disturbances [16]. Disturbance had the smallest net-effect on density in species with a high growth rate, suggesting that migration from undisturbed patches was high in these species, irrespective of their actual swimming speed. In isolated, disturbed communities, recovery from disturbance could by definition only occur by within-population recovery, because no immigration occurred. Since we did not find a correlation between population recovery and intrinsic growth rate (Fig. 7B) in these patches, we infer that population recovery from disturbances largely depended on the influx of individuals from undisturbed patches. This is also supported by the correlation between the predicted rate of spread and recovery rates. However, since our results are correlative and disturbance did not kill all individuals in a patch, we cannot completely separate the recovery effect of within-population growth and immigration. Because species with a high carrying capacity in undisturbed patches recovered much better in the connected disturbed patches (Fig. 7J), we think that the numerical dominance of these species in undisturbed patches may have made emigration to disturbed patches more likely through a direct mass-effect [2]. In other words, more individuals likely created more dispersers even with a constant per capita dispersal rate. Also, dispersal could be density-dependent [5] as reported by recent work for the two protozoan species *Tetrahymena pyriformis* and *Dileptus* sp. [57]. Subsequent fast population growth (of species with a high intrinsic growth rate) after immigration may speed up population recovery from disturbances. In summary, the observed population recovery in disturbed, connected populations in our experiment, is consistent with a temporal storage effect (which occurs when species with a high intrinsic growth rate benefit from the environmental variation caused by recurrent disturbances), and a spatial storage effect, which occurs when species with an high dispersal ability benefit from variation in the occurrence of habitat disturbances across landscapes [58], [59].

Contrary to some theoretical expectations [4], [5], we found no effect of local disturbances on the community composition in adjacent, undisturbed patches, from which net-migration into the disturbed patches occurred. Previous modelling work showed that a cost of emigration may occur when emigration decreases the finite growth rate which may then increase the risk of source extinction [5]. Such an effect has been found in an empirical study of voles [60]. In our experiment, however, species richness and density in undisturbed communities connected to a disturbed patch was neither different from undisturbed communities connected to an undisturbed patch, nor different from undisturbed isolated communities (Figs. 2 and 4; treatment A1, B1 and C1). With our setting, we would have detected a numerical decrease in source populations several times smaller than the observed corresponding increase in population densities in disturbed patches. We therefore conclude that the observed positive effect of connectivity was caused by a combination of immigrating individuals and subsequent population growth, and not only by their direct numerical contribution by migration from undisturbed patches, since this would have caused a large population decrease in the undisturbed source patches. A possible explanation for this finding is that all eight protozoa and rotifer species used in our experiment reproduce asexually, and disturbances were—relative to the generation times—widely spaced. Thus, each individual can contribute equally to a subsequent population growth, either in its source population (when it does not migrate) or in the population it migrated to. Our results are highly consistent over the eleven days between the first and second sampling (Fig. 4 and table 1). Since this time-period not only included two additional disturbance events, but also multiple generations for all protozoa and rotifer species (at least >20), we conclude that the results are robust and meaningful on ecological time-scales, and do not only reflect a highly specific set-up of microcosm experiments [61], [62].

In many empirical and theoretical studies, local disturbances completely kill the local population or community [34], [63]. However, our local disturbances reduced local populations, but did not necessarily kill all individuals in a local patch, and thus had a less severe effect on community composition. A local disturbance did not cause a significant reduction in species richness, and isolated disturbed and undisturbed communities had virtually the same number of species (Fig. 3A, treatment A1 vs. A2). This was somewhat surprising, since many studies using microcosms found a reduction in species richness at similar disturbance intensities [30], [43]. Possibly, in our metacommunities, the connecting tubing was not only a dispersal corridor, but also acted as a refuge for species to persist intense disturbances [31]. While individuals could survive a local disturbance in our experiment, there were no environmental signals preceding a disturbance, which could have prompted individuals to disperse and evade the disturbance. Thereby, only post-disturbance processes such as different growth rates or resource-exploitation as well as active dispersal from undisturbed communities into disturbed communities were likely to occur.

Our study highlights that the effects of patch connectivity and dispersal on systems subject to dispersal are complex. Small amounts of immigration post-disturbance may greatly alter the subsequent densities of species in disturbed communities. Our study also suggests that there is a positive effect of connectivity on population recovery in disturbed patches, even when disturbances do not drive species to extinction, a concept that can be relevant in conservation biology.
